# SARS-CoV-2 Spike Protein Induces Degradation of Junctional Proteins That Maintain Endothelial Barrier Integrity

**DOI:** 10.3389/fcvm.2021.687783

**Published:** 2021-06-11

**Authors:** Somasundaram Raghavan, Divya Borsandra Kenchappa, M. Dennis Leo

**Affiliations:** Department of Pharmaceutical Sciences, University of Tennessee Health Science Center, Memphis, TN, United States

**Keywords:** endothelial barrier function, SARS-CoV-2, angiotensin converting enzyme-2, spike, junctional proteins

## Abstract

Severe acute respiratory syndrome coronavirus 2 (SARS-CoV-2) uses the Angiotensin converting enzyme 2 (ACE2) receptor present on the cell surface to enter cells. Angiotensin converting enzyme 2 is present in many cell types including endothelial cells, where it functions to protect against oxidative damage. There is growing evidence to suggest that coronavirus disease (COVID-19) patients exhibit a wide range of post-recovery symptoms and shows signs related to cardiovascular and specifically, endothelial damage. We hypothesized that these vascular symptoms might be associated with disrupted endothelial barrier integrity. This was investigated *in vitro* using endothelial cell culture and recombinant SARS-CoV-2 spike protein S1 Receptor-Binding Domain (Spike). Mouse brain microvascular endothelial cells from normal (C57BL/6 mice) and diabetic (db/db) mice were used. An endothelial transwell permeability assay revealed increased permeability in diabetic cells as well as after Spike treatment. The expression of VE-Cadherin, an endothelial adherens junction protein, JAM-A, a tight junctional protein, Connexin-43, a gap junctional protein, and PECAM-1, were all decreased significantly after Spike treatment in control and to a greater extent, in diabetic cells. In control cells, Spike treatment increased association of endothelial junctional proteins with Rab5a, a mediator of the endocytic trafficking compartment. In cerebral arteries isolated from control and diabetic animals, Spike protein had a greater effect in downregulating expression of endothelial junctional proteins in arteries from diabetic animals than from control animals. In conclusion, these experiments reveal that Spike-induced degradation of endothelial junctional proteins affects endothelial barrier function and is the likely cause of vascular damage observed in COVID-19 affected individuals.

## Introduction

Severe acute respiratory syndrome coronavirus 2 (SARS-CoV-2) which causes coronavirus disease (COVID-19), is a novel coronavirus that was first reported in China in December 2019 and has since become a pandemic affecting nearly all countries of the world (1–3). Severe acute respiratory syndrome coronavirus 2 patient symptoms range from life-threatening to mild and asymptomatic, which presents unique problems in identifying, quarantining, and treating affected individuals (3). Severe acute respiratory syndrome coronavirus 2 infection leads to “acute respiratory distress syndrome (ARDS)” and respiratory failure (4–7), however, overwhelming evidence suggests that the virus infects other organ systems leading to the coinage of the term “Long COVID” (1, 8–11). Symptoms of cardiovascular dysfunction has been observed in many COVID patients and recovered individuals and the mechanisms behind these symptoms have not been adequately explored. Additionally, patient data analysis has also revealed that the highest-risk for severe COVID-19 or death is amongst patients that have pre-existing conditions specifically, hypertension, obesity, and type 2 diabetes (T2D) (12). The relationship between these pre-existing conditions and COVID-19 is also unclear.

Angiotensin converting enzyme 2 (ACE2) is a type I membrane-localized glycoprotein highly expressed in lung alveolar cells, vascular endothelial cells, cardiac myocytes, and various other cell types and is the primary receptor used by SARS-CoV-2 for cellular entry (13). The SARS-CoV-Spike (S) protein is one of four virus structural proteins made up of S1 and S2 subunits of which the S1 subunit contains the “Receptor Binding Domain” (RBD) which mediates binding to cell surface ACE2 (14, 15). Endothelial cell barrier function is mediated by three types of “junctional” proteins, adherens, tight, and gap junctions (16–18). Junctional protein function is tightly regulated under normal conditions to aid in immune response, metabolite exchange, and wound healing (17, 18). Disruption in the expression of these proteins can lead to impaired endothelial barrier function and wider cardiovascular complications. We hypothesized that the SARS-CoV-2 spike protein binds to the ACE2 receptor in endothelial cells leading to its internalization and degradation and in doing so, also triggers the downregulation of critical proteins that maintain endothelial barrier function leading to compromised barrier integrity which could initiate further cardiovascular damage.

Our results show that in control cells and in healthy mouse cerebral arteries, spike protein triggered downregulation of critical junctional proteins, VE-Cadherin, PECAM-1, JAM-A, and Connexin-43. The expression of these proteins appeared to be downregulated in diabetic cells and diabetic mouse cerebral arteries when compared to control but spike induced a further reduction in protein expression. Co-immunoprecipitation experiments showed that in control cells, spike treatment increased association of ACE2, PECAM-1, JAM-A, and Connexin-43 with the endocytic trafficking protein, Rab5a. An endothelial permeability assay also revealed increased permeability in diabetic cells which further increased in the presence of spike protein. These early results showed that spike protein not only binds to endothelial ACE2 to induce its degradation but also likely triggered additional dysfunction by downregulation of endothelial junctional proteins.

## Materials and Methods

### Cell Culture

C57BL/6 and *db/db* diabetic mice Primary Brain Microvascular Endothelial Cells were purchased from Cell Biologics and cultured in Endothelial Cell Medium containing (0.5 ml VEGF, ECGS, Heparin, EGF, Hydrocortisone, L-Glutamine, Antibiotic-Antimycotic Solution) supplemented with 2% fetal bovine serum.

### Reagents

Severe acute respiratory syndrome coronavirus 2 (COVID-19) S1 Recombinant Protein (cat. no. 97-092) was purchased from ProSci Inc (Poway, CA) and used at a final concentration of 10 μg/ml. Cells or isolated arteries were treated with Spike protein at the final concentration for 12 h before being used for experiments. Anti-VE-Cadherin antibody (cat. no. NBP1-43347), Anti-PECAM1 Antibody (cat. no. NB600-562) were purchased from Novus Biologicals (Centennial, CO). Anti-Jam-A Antibody (cat. no. 14-3219-82) were purchased from Invitrogen (Carlsbad, CA). Anti-Connexin-43 Antibody (cat. no. 3512), Anti-Rab5a Antibody (cat. no. 46449), antibodies were procured from Cell Signaling Technology (Danvers, MA). Anti-ACE2 Antibody (cat. no. PAB13443) were purchased from Abnova (Taipei, Taiwan). Anti-Actin Antibody (#MAB1501) were obtained from Millipore (Burlington, MA).

### Animals

All procedures were approved by the Animal Care and Use Committee of the University of Tennessee. All experiments were performed using C57BL/6J mice (12 weeks) unless otherwise stated. C57BL/6J mice were purchased from Jackson Laboratories. Male C57BL/6J mice were maintained on a High Fat Diet (HFD) from 6 weeks of age until the end of the treatment period at 24 weeks. This model has been detailed in a recently published study from our lab (19).

### Endothelial Cell Permeability Assay

The Endothelial Transwell Permeability Assay Kit (Cell Biologics, Inc., Chicago, IL) was used as per manufacturer's instructions. Briefly, control and diabetic cells were grown to confluence and HRP absorbance values were recorded from untreated and Spike treated cell chambers. Data was normalized to untreated control cells and expressed as fold change.

### Cerebral Artery Isolation

Mice were euthanized with isoflurane (1.5 %) followed by decapitation. Cerebral (anterior, middle and posterior cerebral and cerebellar) arteries were removed and placed into ice-cold physiological saline solution (PSS) that contained (in mM): 112 NaCl, 6 KCl, 24 NaHCO_3_, 1.8 CaCl_2_, 1.2 MgSO_4_, 1.2 KH_2_PO_4_, and 10 glucose, gassed with 21% O_2_, 5% CO_2_, and 74% N_2_ to pH 7.4. During Spike treatment, arteries were maintained in DMEM F-12 media supplemented with penicillin/streptomycin without FBS and in a regular cell culture chamber. Arteries were cleaned and minced artery pieces were homogenized in cold lysis buffer in a glass dounce homogenizer. The homogenates were centrifuged at 15,000 g for 15 min at 4°C. Protein concentrations were determined with the BCA protein assay kit (Pierce Chemical, Rockford, IL).

### Western Blotting

Arterial segments were pooled from between two and three mice for experiments measuring protein abundance. Western blotting for total protein was done following standard protocols. Proteins were separated on 7.5% SDS-polyacrylamide gels and transferred onto nitrocellulose membranes. Membranes were blocked with 5% non-fat milk and incubated with the one of the following primary antibodies: VE-Cadherin, PECAM1 (Novus Biologics), JAM-A (Invitrogen), Connexin-43, Rab5a (Cell Signaling Inc.), ACE2 (Abnova), or Actin (Millipore Sigma) overnight at 4°C. Membranes were washed and incubated with horseradish peroxidase conjugated secondary antibodies at room temperature. In some cases, blots were physically cut to allow for probing of two different proteins without the need for stripping. Protein bands were imaged using a ChemiDoc gel imaging system, quantified using Quantity One software (Biorad), and normalized to actin.

### Immunoprecipitation

After rinsing with cold phosphate-buffered saline (PBS), cells were lysed in 350 ml of lysis buffer. The cell extracts were cleared by centrifugation at 10,000 rpm for 10 min at 4°C. The cleared cell extracts containing an equal amount of protein (150 μg) from control and the indicated treatments from lysed cells was used for immunoprecipitation using a commercially available kit (Catch and Release version 2.0 Reversible Immunoprecipitation System, catalog no. 17–500) according to the manufacturer's specification (Millipore Inc.). In brief, cell lysate (150 μg) with ACE2 or Rab5a antibody (2 μg), antibody capture affinity ligand (that binds the antibody–antigen complex and tethers it to a resin), were mixed in microcentrifuge spin columns (18 h at 4°C). Subsequently, unbound proteins were removed by centrifugation, and captured antibody–antigen complex was eluted by heating the beads in 40 μl of Laemmli sample buffer and analyzed by Western blotting for the indicated molecules using their specific antibodies.

### Statistics

Statistical analysis was performed using OriginLab and GraphPad InStat software. Data are shown as means ± SE and expressed as fold change normalized to respective actin and untreated controls. Mann–Whitney *U*-test, and ANOVA with Bonferroni's *post-hoc* test for multiple group comparisons were used where appropriate. *P* < 0.05 was considered significant.

## Results

### Spike Increases Endothelial Barrier Permeability

**Figure 1:**
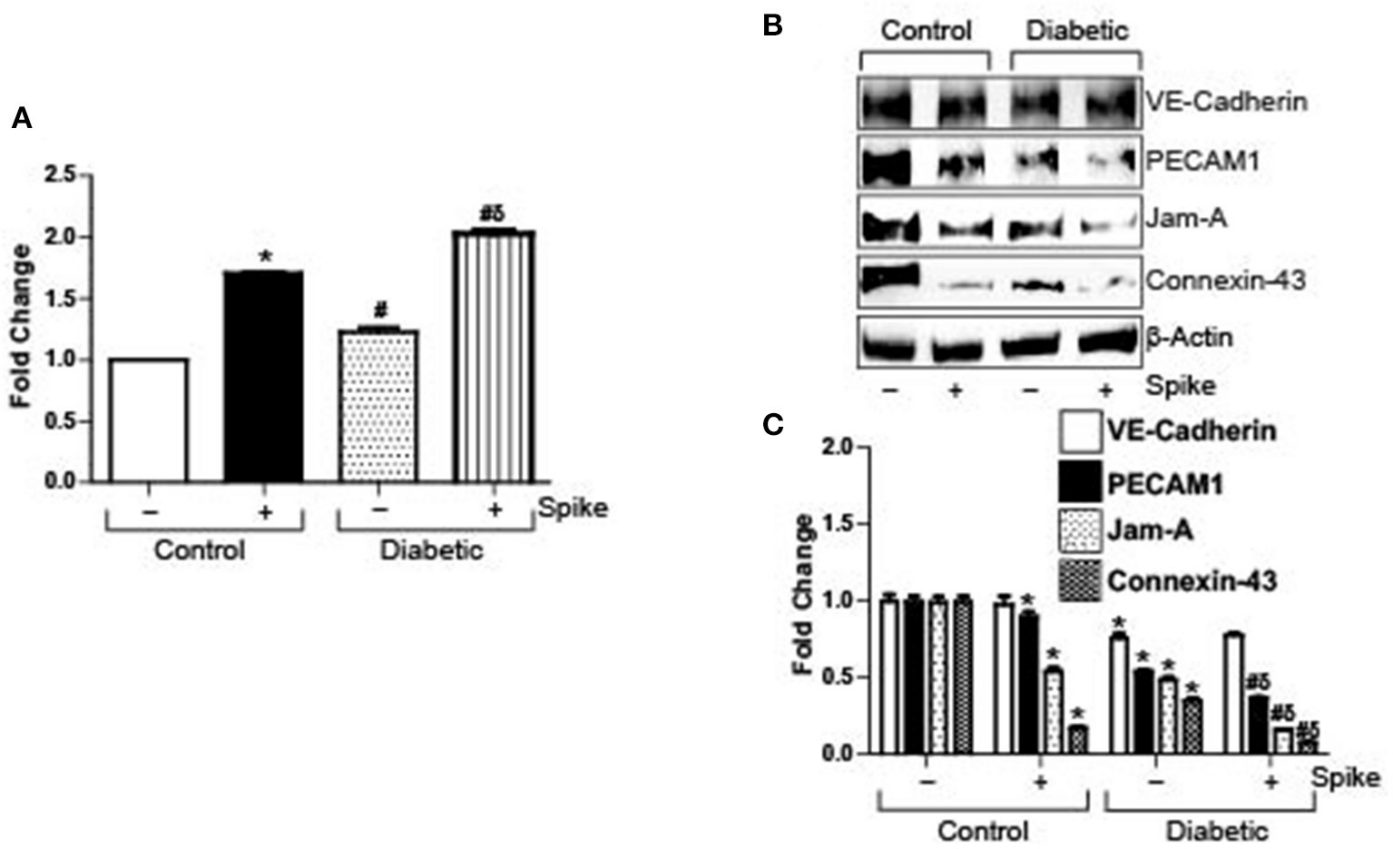
SARS-CoV-2 spike protein (Spike) increases endothelial permeability by downregulation of junctional proteins in diabetic endothelial cells. **(A)** Endothelial transwell permeability assay, mean data expressed as fold change. *n* = 6 for each, *P < 0.05 vs. untreated control, ^δ^*P* < 0.05 vs. untreated diabetic, ^#^*P* < 0.05 vs. Spike-treated control. **(B)** Representative Western blot indicating expression of endothelial junctional proteins in endothelial cell culture with or without Spike treatment. **(C)** Mean data indicating fold change in protein expression. *n* = 6 for each, *P < 0.05 vs. untreated control, ^δ^*P* < 0.05 vs. untreated diabetic, ^#^*P* < 0.05 vs. Spike-treated control.

Severe acute respiratory syndrome coronavirus 2 (SARS-CoV-2) causes a host of pathological conditions that indicate compromised vascular function. To investigate if internalization of the ACE2 receptor by Spike alone triggered this response, brain microvascular endothelial cells were seeded to confluency and endothelial permeability was assayed using an Endothelial transwell permeability assay kit. Results indicate that after 12 h of 10 μg/ml Spike treatment, endothelial permeability increased ~1.8-fold in control cells (Figure 1A). In contrast, untreated diabetic cells showed ~1.2-fold more endothelial permeability compared to untreated controls (Figure 1A). After Spike treatment however, diabetic endothelial cell permeability increased ~2-fold compared to untreated controls and was significantly greater than Spike treated control endothelial cells (Figure 1A). These results indicate that Spike alone was able to induce disruption of endothelial barrier integrity in control cells. Expectedly, these results also show that diabetic endothelial barrier permeability is affected in untreated cells and that Spike induced an even greater disruption of endothelial barrier function.

### Spike Differentially Affects the Expression of Endothelial Junctional Proteins

Spike binds to ACE2 receptor present on the cell surface of different cells to induce internalization and degradation of the protein triggering virus cellular entry. In the absence of a complete virion, we wanted to investigate if Spike-induced ACE2 internalization and degradation was the primary reason for the failing endothelial barrier integrity. Protein expression levels of different types of endothelial junctions were analyzed after 12 h of 10 μg/ml Spike treatment or without treatment in both normal and diabetic endothelial cells. Results indicate that expression of the adherens junction protein, VE-Cadherin, was decreased after Spike treatment (Figures 1B,C). PECAM-1 expression also showed a small but significant decrease after spike treatment in control cells (Figures 1B,C). Similarly, the tight junction protein JAM-A, and the gap junction protein Connexin-43 decreased by ~50 and 80%, respectively, in the presence of Spike when compared to untreated controls (Figures 1B,C).

In untreated diabetic endothelial cells, expression of VE-Cadherin, PECAM-1, JAM-A, and Connexin-43 were significantly lower compared to untreated control cells (Figures 1B,C). However, in the presence of Spike, except for VE-Cadherin, the expression of all proteins further decreased significantly, with maximal decrease observed with JAM-A and Connexin-43 expression levels (Figures 1B,C). These results indicate that Spike induces differential effects on endothelial junctional proteins to affect barrier function.

### Spike Induces Internalization of Endothelial ACE2 and Junctional Proteins Through a Rab5-Mediated Pathway

**Figure 2:**
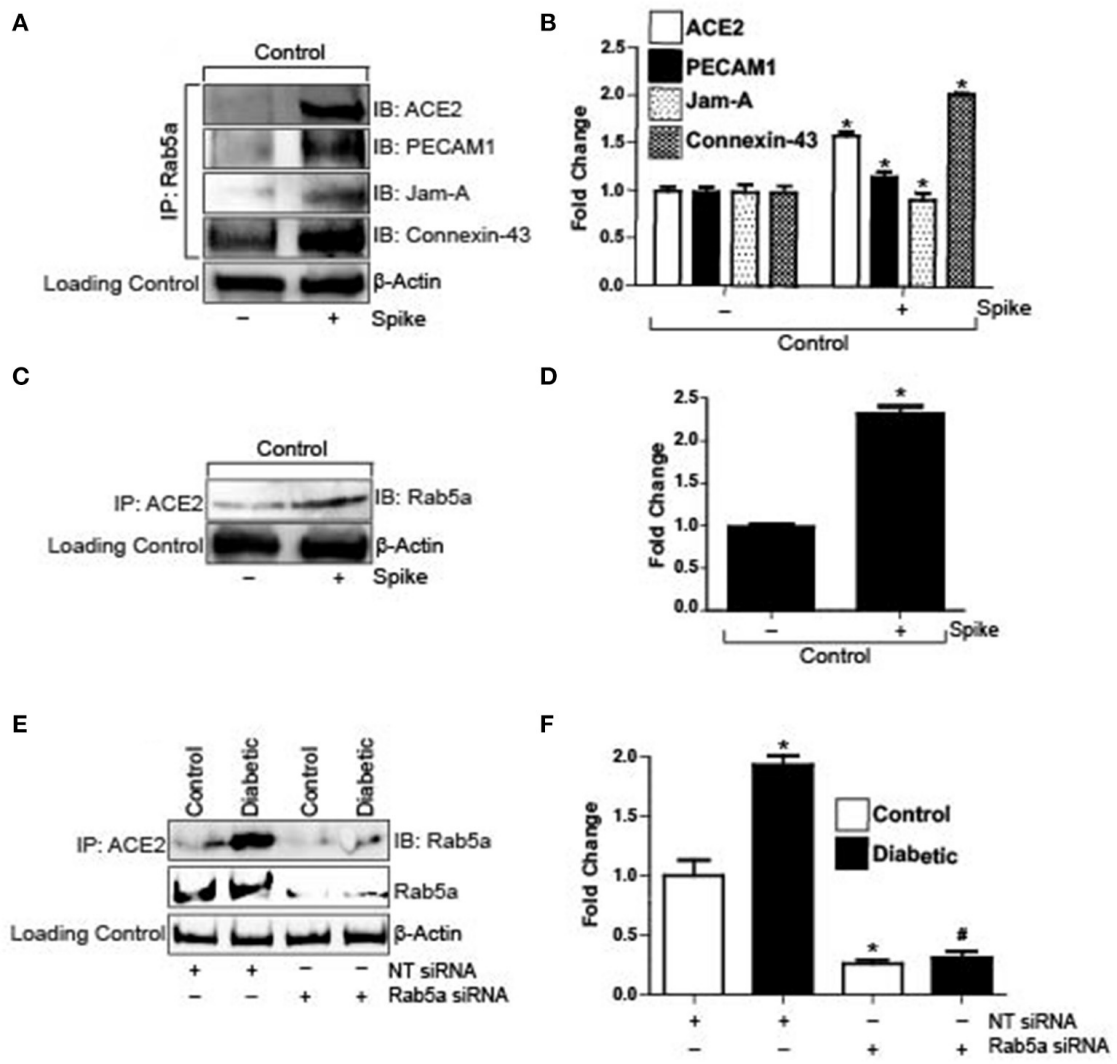
Spike increases Rab5a association with ACE2 and junctional proteins. **(A)** Representative Western blot of coimmunoprecipitation experiments done using Rab5a antibody and probed for different proteins. **(B)** Mean data indicating fold change in Rab5a association with junctional proteins and ACE2 after S1RBD treatment. *n* = 6 for each, *P < 0.05 vs. untreated control. **(C)** Representative Western blot of coimmunoprecipitation experiments done using ACE2 antibody and probed for Rab5a. **(D)** Mean data indicating fold change in ACE2 association with Rab5a after Spike treatment. *n* = 6 for each, *P < 0.05 vs. untreated control. **(E)** Representative Western blot of coimmunoprecipitation experiments in control and diabetic cells after Rab5a knockdown with siRNA. **(F)** Mean data indicating fold change in ACE2 association with Rab5a after Rab5a siRNA. *n* = 6 for each, *P < 0.05 vs. control, ^#^*P* < 0.05 vs. NT siRNA diabetic.

Severe acute respiratory syndrome coronavirus 2 (SARS-CoV-2) enters cells when its spike protein-RBD binds to ACE2 on the cell surface, which subsequently causes internalization of the receptor and the virion. To investigate the possible entry mechanisms of Spike, we performed semi-quantitative co-immunoprecipitation in control cells with and without Spike treatment. Results indicate that in untreated cells, ACE2, PECAM-1, and JAM-A did not immunoprecipitate with Rab5a, a key Rab GTPase that regulates the endolysosomal trafficking pathway (Figures 2A,B). Interestingly, Connexin-43 immunoprecipitated with Rab5a in untreated cells (Figures 2A,B). However, in the presence of Spike, Rab5a immunoprecipitated with ACE2 and the junctional proteins, PECAM-1 and JAM-A (Figures 2A,B). Association of Rab5a with Connexin-43 increased ~2-fold when compared with untreated cells (Figures 2A,B). These results indicate that Spike induced internalization of ACE2, PECAM-1, JAM-A, and Connexin-43 through a Rab5-dependent endocytic pathway. Immunoprecipitation was also performed using the ACE2 antibody and the blots probed for Rab5a. Results indicate that in untreated cells, only a small percentage of ACE2 is associated with Rab5a (Figures 2C,D). In contrast, there was a 2-fold increase in ACE2 association with Rab5a after spike treatment, indicating that spike activates a Rab5-mediated pathway to internalize ACE2. To further probe association of Rab5 with ACE2, cells were treated with Rab5a siRNA for 24 h and pulldown experiments were repeated. After knockdown of Rab5a, there was no difference in Rab5a pulldown between control and diabetic cells (Figures 2E,F) which confirmed that there was increased association of ACE2 with Rab5a in diabetic cells.

### Spike Triggers Greater Decrease in Endothelial Junctional Proteins in Diabetic Mouse Cerebral Arteries

**Figure 3:**
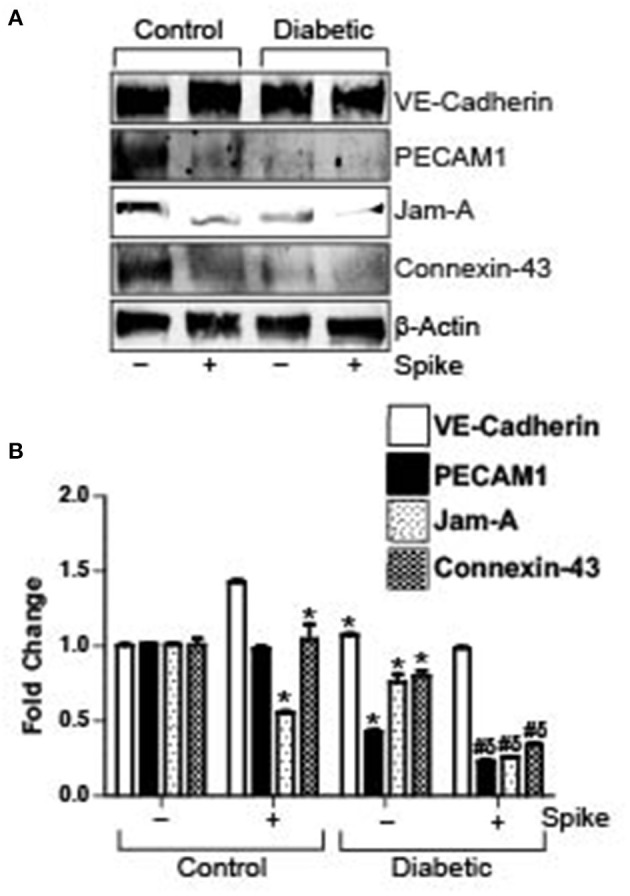
Spike-induced degradation of endothelial junctional proteins is greater in arteries of diabetic mice. **(A)** Representative Western blot indicating expression of endothelial junctional proteins in arteries isolated from control or diabetic mice. **(B)** Mean data indicating fold change in protein expression. *n* = 4 for each, *P < 0.05 vs. untreated control, ^δ^*P* < 0.05 vs. untreated diabetic, ^#^*P* < 0.05 vs. Spike-treated control.

Diabetes is associated with a decrease in endothelial barrier function. In our high fat-fed diabetic mice there was noted reduction in expression of VE-Cadherin, PECAM-1, JAM-A, and Connexin-43 in diabetic mouse arteries as compared to untreated control arteries. Treatment of control arteries with spike decreased PECAM-1, JAM-A, and Connexin-43 levels but not VE-Cadherin. Similarly, there was significant decrease in these proteins, except for VE-Cadherin in diabetic arteries treated with spike (Figures 3A,B). These results indicate that existing dysfunctional endothelial barrier function in diabetic cerebral arteries is further increased in the presence of spike protein.

Taken together our results show that Spike-induced ACE2 internalization in endothelial cells likely triggers changes in endothelial cell physiology that leads to internalization and degradation of key junctional proteins to disrupt endothelial barrier integrity and cell communication.

## Discussion

In this study, we investigated the hypothesis that SARS-CoV-2 spike protein internalization causes disruption of brain microvascular endothelial barrier function and intercellular signaling by downregulating critical proteins and that diabetic endothelial cells are more susceptible to these effects. Our results indicated that in healthy cells treated with spike protein, VE-Cadherin expression was unaffected, but expression of PECAM-1, JAM-A, and Connexin-43 significantly decreased. An endothelial transwell permeability assay also indicated significant increase in endothelial permeability after spike treatment. In contrast, untreated diabetic endothelial cells showed decreased expression of all the above-mentioned proteins when compared to controls and that expression of these proteins except for VE-Cadherin, were greatly reduced after spike treatment. In control cells, spike treatment increased association of ACE2 with the retrograde trafficking protein Rab5a, as did PECAM-1, JAM-A, and Connexin-43. Similar protein expression patterns were also observed from cerebral arteries obtained from control and diabetic mice. Taken together these results indicated that internalization of spike protein after binding to ACE2 was primarily mediated by a Rab5-dependent internalization pathway and that this process triggered the downregulation of several proteins that are vital for normal endothelial function.

Endothelial cell barrier function is significantly affected in several diseases, including diabetes, cancer, and chronic inflammation, which can lead to life-threatening consequences. The primary route of entry for SARS-CoV-2 are the airway epithelial cells, after which it migrates to the lung alveolar epithelial cells, where it rapidly replicates and triggers a massive immune response called the “cytokine storm,” leading to “ARDS” and respiratory failure (4–7). The surface receptor that SARS-CoV-2 uses for cellular entry, the ACE2, is widely present in all cell types, including endothelial cells (EC) of small resistance arteries (20–22). Available evidence suggests COVID-19 causes severe vascular damage by disrupting endothelial function (1, 9–11). The disease is now also associated with brain edema, inflammation, hemorrhage and memory loss (8), all of which point to dysfunctional cerebral vascular function.

Microvascular endothelial cells express three types of junctional proteins, adherens junctions (AJ), tight junctions (TJ), and/or gap junctions (GJ). VE-Cadherin is a critical regulator of AJ function. In our study, the expression of this protein was significantly reduced in diabetic endothelial cells and arteries, but it was surprising that spike protein did not induce a further decrease in protein expression given that we found an increase in endothelial permeability after spike treatment. While many previous studies have found significant decrease in VE-Cadherin expression in cultured endothelial cells in response to bacterial-LPS treatment, a few have also reported that expression did not change after LPS challenge (23). VE-Cadherin is a predominantly membrane-localized protein (23, 24) and after LPS challenge it was internalized and degraded, thereby affecting barrier permeability (23). However, it has also been suggested that VE-Cadherin could be internalized within the endothelial cell but not degraded (23). Cytoplasm-localized VE-Cadherin would not be able to function to main barrier integrity thereby increasing permeability, as indicated by our permeability assay results. Techniques like Western Blotting might be insufficient to delineate this difference in protein localization and function.

JAM-A is a tight junction protein which controls cell polarity, barrier function, and leukocyte recruitment (18). JAM-A is highly expressed in brain vasculature where it contributes to TJ function (18). PECAM-1 is an adhesion molecule enriched at endothelial junctions of microvessels and mediates neutrophil diapedesis across the arterial wall as well as serving to maintain vascular integrity (25, 26). JAM-A gene knockouts showed increased endothelial permeability while TNFα and IFNγ treatment in HUVECs decreased PECAM-1 mRNA and protein expression (27, 28). The lateral border recycling compartment (LBRC) is a unique endothelial specific compartment involved in internalization and recycling or degradation of several important junctional proteins (17). PECAM-1 and JAM-A but not VE-Cadherin can undergo internalization and degradation through this compartment (29). Vascular and systemic inflammation mediates changes in expression patterns of these proteins to aid in the response to an insult and mediate the healing process (16–18). Connexin-43 is a GJ protein that mediates cell–cell communication by exchange of ion, small metabolites, and even signaling molecules (30). Inflammatory mediators like LPS, TNFα, and thrombin are all known to decrease Connexin-43 expression (30). Our investigation has shown that spike protein significantly decreased expression of these proteins in control cells and arteries and that its effect was further augmented in diabetic cells and arteries. These results indicate that there is loss of barrier integrity and intercellular communication after spike protein challenge.

Evidence that COVID-19 causes vascular damage emerged early on during the pandemic and it is now regarded as an important adverse outcome caused by the disease (31, 32). “Long COVID” is a term that has recently evolved to describe the wide variety of symptoms that appear in recovered patients. Several “long COVID-19” symptoms point to significant vascular damage even in individuals who were asymptomatic or had mild symptoms (1, 8–11). Our results here reveal a significant impact on normal endothelial function. However, the more interesting facet with these results was that the experiments were conducted *in vitro* and using a recombinant protein and does not involve any mediators that would have been present from a systemic immune response. Unlike other inflammatory mediators, including various cytokines, that have their own cell surface receptors and intracellular signaling networks, spike protein binds to ACE2 on the cell surface to induce its internalization and degradation through a Rab5-mediated pathway, but downstream intracellular signaling networks that this event initiates remains unclear (33, 34). Endothelial cells are known to be able to produce their own pro-inflammatory mediators, and it has also recently emerged that these cells might have a greater role in the outcomes of many viral diseases (35, 36). Results here show that the ACE2-spike complex being internalized and degraded, but this alone does not explain the loss in junctional proteins that are vital for vascular function, since there were no other viral components or systemic inflammatory mediators present. It is likely that processes that regulate endothelial ACE2-spike internalization and degradation also lead to an increase in synthesis and secretion of pro-inflammatory mediators from the endothelial cells themselves that possibly accelerates barrier disruption. This phenomenon would need to be investigated in further detail, but our early data has provided evidence that endothelial cells could be important mediators of both the “cytokine storm” and “long COVID” symptoms.

## Conclusion

Vascular damage from COVID-19 has once again come to the forefront with the emergence of “long COVID” symptoms. The data presented here suggests that endothelial cells likely play a larger role in initiating and sustaining the vascular damage observed in COVID-19 patients and survivors.

## Data Availability Statement

The original contributions generated for the study are included in the article/supplementary materials, further inquiries can be directed to the corresponding author/s.

## Ethics Statement

The animal study was reviewed and approved by Animal Care and Use Committee of the University of Tennessee.

## Author Contributions

MDL conceived, designed research, drafted manuscript, and edited and revised manuscript. SR and DK performed western blotting, coimmunoprecipitation experiments, and analyzed data. MDL, SR, and DK interpreted results of experiments and approved final version of manuscript. SR prepared figures. All authors contributed to the article and approved the submitted version.

## Conflict of Interest

The authors declare that the research was conducted in the absence of any commercial or financial relationships that could be construed as a potential conflict of interest.
